# ClearF++: Improved Supervised Feature Scoring Using Feature Clustering in Class-Wise Embedding and Reconstruction

**DOI:** 10.3390/bioengineering10070824

**Published:** 2023-07-10

**Authors:** Sehee Wang, So Yeon Kim, Kyung-Ah Sohn

**Affiliations:** 1 Department of Artificial Intelligence, Ajou University, Suwon 16499, Republic of Korea; wsh0509@ajou.ac.kr (S.W.); jebi1771@ajou.ac.kr (S.Y.K.); 2 Department of Software and Computer Engineering, Ajou University, Suwon 16499, Republic of Korea

**Keywords:** feature selection, feature scoring, information theory, entropy, mutual information (MI), dimension reduction, low-dimensional embedding, reconstruction error, principal component analysis (PCA), clustering

## Abstract

Feature selection methods are essential for accurate disease classification and identifying informative biomarkers. While information-theoretic methods have been widely used, they often exhibit limitations such as high computational costs. Our previously proposed method, ClearF, addresses these issues by using reconstruction error from low-dimensional embeddings as a proxy for the entropy term in the mutual information. However, ClearF still has limitations, including a nontransparent bottleneck layer selection process, which can result in unstable feature selection. To address these limitations, we propose ClearF++, which simplifies the bottleneck layer selection and incorporates feature-wise clustering to enhance biomarker detection. We compare its performance with other commonly used methods such as MultiSURF and IFS, as well as ClearF, across multiple benchmark datasets. Our results demonstrate that ClearF++ consistently outperforms these methods in terms of prediction accuracy and stability, even with limited samples. We also observe that employing the Deep Embedded Clustering (DEC) algorithm for feature-wise clustering improves performance, indicating its suitability for handling complex data structures with limited samples. ClearF++ offers an improved biomarker prioritization approach with enhanced prediction performance and faster execution. Its stability and effectiveness with limited samples make it particularly valuable for biomedical data analysis.

## 1. Introduction

In the field of bioinformatics, accurate disease classification is crucial for effective diagnosis and treatment. Furthermore, the precise identification and selection of relevant biomarkers is essential to predict disease risk or aid in drug development [1]. As a result, a significant amount of research is currently being conducted in biomarker detection. Feature selection methods [2,3,4] are widely used in this context to identify and prioritize biomarkers from large and complex datasets [5,6]. These methods are particularly valuable in bioinformatics, where datasets often have a high number of features relative to the number of samples. By reducing the dimensionality of such data, feature selection can help identify the most informative biomarkers, facilitating accurate disease classification. Many feature selection algorithms have been developed to precisely select the most relevant biomarkers. This is crucial to better understand the underlying mechanisms of disease development and prognosis and to develop more targeted therapies.

Feature selection methods can be broadly classified into supervised and unsupervised approaches, where supervised approaches utilize class labels to identify relevant features, while unsupervised approaches do not [7]. As supervised approaches are more suitable for targeting specific diseases and finding relevant biomarkers, this study utilizes supervised feature selection methods for effective biomarker prioritization. There are various supervised feature selection methods, such as statistical methods [8,9], similarity-based approaches [10,11], and information-theoretic methods. Information-theoretic methods perform feature selection by quantifying the amount of mutual information, which is a measure of entropy and conditional dependencies between data variables and their labels. Information-theoretic methods have been widely studied for feature selection, and their effectiveness has been demonstrated by promising experimental results [12,13,14,15,16]. Recently, innovative approaches like MI-VIF [17] have emerged, which combine variance inflation factor and mutual information, offering a solution to the collinearity problem that leads to unstable parameter estimation. In addition, a methodology named Relevance based on Weight Feature Selection (RWFS) [18] has been proposed. This method is based on two types of changed ratios in relation to feature relevance evaluation: one for the undetermined amount of information and the other for the established amount of information. These strategies have demonstrated their effectiveness by improving performances

However, these methods often suffer from high computational costs, and they may require discretization of continuous variables, which may lead to information loss [19]. To address these issues, we have previously proposed ClearF [20], which uses the reconstruction error of a low-dimensional embedding method as a proxy for the mutual information. ClearF assigns supervised scores to features by applying unsupervised class-wise low-dimensional embedding, which has been demonstrated to be effective in several benchmark datasets. However, ClearF has a limitation in that the selection process of the bottleneck layer is not transparent, requiring the selection of feature size in advance, followed by a greedy search. Consequently, the process can be complicated, unstable, and time-consuming, depending on the experimental setup. Furthermore, due to the partitioning of the entire dataset based on class labels and the subsequent embedding of each partition, the sample size becomes significantly smaller. This may introduce the risk of generating unstable outcomes during the feature selection process.

**Figure 1:**
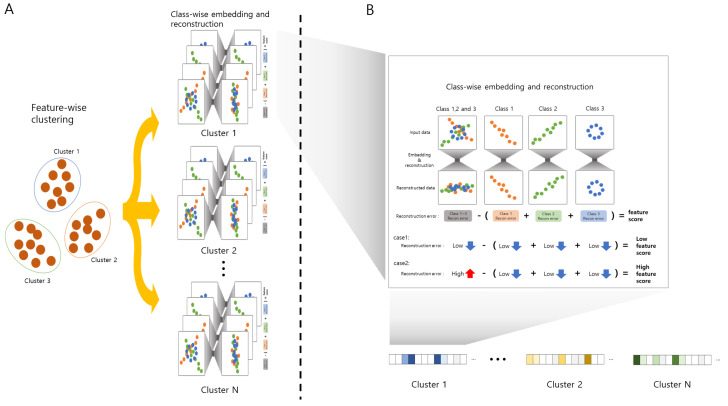
Overview of ClearF++, a supervised feature scoring method that utilizes feature clustering in a class-wise embedding and reconstruction method: (**A**) Description of how the entirety of the data are divided into multiple partitions using feature-wise clustering. (**B**) Description of the process of calculating feature importance using ClearF++.

In this paper, we propose ClearF++ to address the limitations of ClearF. ClearF++ simplifies the process of determining the number of uncertain bottleneck layers and further improves performance through feature clustering. First, we propose a method to increase convenience and stabilize the process by simply fixing the number of bottleneck layers to a single value. In addition, we apply a feature-wise clustering method to mitigate the problem of embedding too many features at once and only reflecting the importance of a few features. This method allows for the selection of important features by clustering similar features together, thus reducing the number of embedded features. In summary, ClearF++ addresses the limitations of ClearF by simplifying the selection process of bottleneck layers and improving performance through feature clustering. Figure 1 illustrates the proposed architecture, and the entire process is shown in the pseudocode presented in Algorithm 1.
**Algorithm 1** Algorithm ClearF++: Supervised feature scoring method using feature clustering in the class-wise embedding and reconstruction method.1:**function** ClearF++(X,Y,k,l)2:      **Input:**      $X=\left\{X_{1},X_{2},\ldots ,X_{s}\right\}\in R^{n\times s}$: Data matrix (n features and s samples)      $Y=\{y_{1},y_{2},\ldots ,y_{s}\}$: Label vector      *k*: Number of clusters      *l*: Number of classes3:      **Output:**      $F=\{f_{1},f_{2},\ldots ,f_{n}\}$: Feature scores4:      Perform feature-wise clustering on data *X*:5:      Apply DEC clustering method that divides n features into k clusters to obtain $C=\{C_{1},C_{2},\ldots ,C_{k}\}$, where each cluster $C_{i}\in R^{c_{i}\times s}$.6:      **for** i=1,…,k **do**7:            $F_{i}\leftarrow$ ClearF++$\left(C_{i},Y,l,1\right)$
8:      **end for**9:      Aggregate feature scores for each cluster and rank features to obtain *F*10:    **return** *F*11:**end function**12:**function** ClearF(X,Y,l,d)13:      **Input:**      $X=\left\{X_{1},X_{2},\ldots ,X_{s}\right\}\in R^{n\times s}$: Data matrix      $Y=\{y_{1},y_{2},\ldots ,y_{s}\}$: Label vector      *l*: Number of classes      *d*: Number of components14:      **Output:**      $F=\{f_{1},f_{2},\ldots ,f_{n}\}$: Feature scores15:      Using label vector *Y*, divide *X* into $L=\{L_{1},L_{2},\ldots ,L_{l}\}$, where each divided data $L_{j}\in R^{n\times l_{j}}$.16:      Perform low-dimensional embedding on *X* with *d* components and reconstruct to calculate the feature-wise reconstruction error:17:      $R_{X}=\left\{r_{\left(X,1\right)},r_{\left(X,2\right)},\ldots ,r_{\left(X,n\right)}\right\}$18:      **for** j=1,…,l **do**19:            Perform low-dimensional embedding on $L_{j}$ and reconstruct to calculate the feature-wise reconstruction error:20:            $R_{j}=\left\{r_{\left(j,1\right)},r_{\left(j,2\right)},\ldots ,r_{\left(j,n\right)}\right\}$21:      **end for**22:      $R_{\mathrm{sum}}=\mathrm{sum}\left(R_{1},R_{2},\ldots ,R_{l}\right)$23:      $F=R_{X}$−$R_{\mathrm{sum}}$24:      **return** *F*25:**end function**


## 2. Materials and Methods

### 2.1. ClearF-One: Simplifying Bottleneck Layer Selection

To tackle the instability issue in the bottleneck layer selection process within ClearF, we propose a refined approach called ClearF-one. In this modified method, the bottleneck layer is set to a single layer rather than employing a greedy search to determine the optimal number of bottleneck layers. As the size of the bottleneck layer increases, a broader range of information is selected, resulting in the dilution of focusing important parts. By constraining the number of layers to one, only the most informative features from each class’s embedding are selected, aligning with the theoretical foundation of ClearF. In summary, ClearF-one serves as an enhanced version of ClearF that addresses instability in the feature selection process by simplifying the bottleneck layer to a single layer.

### 2.2. ClearF++: Advanced Feature Selection via Feature-Wise Clustering

As described above, ClearF-one fixes the bottleneck layer to a single layer, resulting in features with strong signals for each class that are likely to have high scores. However, ClearF-one can be disadvantageous in selecting multiple features due to the limited amount of expressed information. To overcome this limitation, we propose a novel method that divides the data into several partitions through feature-wise clustering and applies ClearF-one to each cluster. As shown in Figure 1A, feature-wise clustering is performed to divide the data into units of each cluster with similar features. When we perform feature-wise clustering, the Deep Embedding Clustering (DEC) method [21] is applied, which is a method of unsupervised learning that combines deep neural networks with clustering algorithms. Next, ClearF-one is applied to each of the clustered data to calculate the feature score. Finally, ClearF++ produces a high feature score when it exhibits a significant difference between classes, such as in case 2 of Figure 1B. Features with no significant difference between classes, such as case 1, are not scored high. This approach allows the most informative features to produce high scores by calculating a class-wise reconstruction error. The above process is performed for each cluster, as depicted in Figure 1B, extracting features that encapsulate important characteristics unique to each cluster.

Our proposed method ClearF++ has several advantages over ClearF and ClearF-one. It is particularly useful when an appropriate number of features must be selected from data with a large number of features, such as in biomarker identification. Additionally, it can be applied when the number of samples is too small compared with the number of features, making it difficult to learn ClearF stably. In summary, ClearF++ divides the data into feature-wise clusters and applies ClearF-one to each cluster, enabling us to select multiple informative features from a large number of features.

## 3. Results

### 3.1. Datasets

We conducted an experiment on the gene expression data of lung cancer patients using the ARCHS4 dataset [22], which has been used in several studies [23,24]. We removed genes that had more than 25% zero expression across all samples. The experiment was tested with 8710 genes and 3079 samples. Out of the 3079 samples, 1158 samples belong to the A549 cells (non-small-cell lung cancer) and 1921 samples belong to the IMR90 cells (normal lung fibroblast). Additionally, we performed experiments on several benchmark datasets. To externally validate our results, we further conducted the experiments over two additional benchmark datasets, colon and ALL/AML leukemia datasets [7]. The ALL/AML dataset consists of 72 samples, 47 samples belong to acute lymphoblastic leukemia (ALL) and 25 samples belong to acute myeloid leukemia (AML) [25]. Gene expression levels were measured using Affymetrix high-density oligonucleotide arrays containing 7129 genes. The colon dataset consists of 62 samples, of which 22 are normal and 40 are colon tumor tissue samples [26]. Gene expression levels were measured using Affymetrix oligonucleotide arrays containing expression levels for the 2000 genes with the highest minimal intensity across the samples, as it is prepared in the paper [26].

### 3.2. Performance Evaluation on Multiple Benchmark Datasets

We compared the performance of several feature selection algorithms, including MultiSURF, IFS, and ClearF, to demonstrate the effectiveness of our proposed method in extracting the most relevant features for lung cancer classification using the ARCHS4 dataset. The selected features from each method were used for classification and their AUCs were compared. We performed 10-fold cross-validation by dividing the entire dataset into 10 folds, with one fold for test data and the remaining folds for training data. Each feature selection algorithm was applied solely to the training data to select important features. A classification algorithm was then applied using only the selected features, and the average AUC of the 10-fold cross-validation was measured. The classification model is a basic four-layer DNN, consisting of an input layer, two hidden layers, and an output layer. The sizes of the hidden layers were determined as the number of selected features * 2 and the number of selected features, respectively. The hyperbolic tangent served as the activation function. The Adam optimizer was employed for learning with a learning rate of 1 × 10${}^{-3}$ and 500 epochs using a full batch.

To validate the stability and effectiveness of our proposed method, ClearF++, we conducted a performance comparison using the ARCHS4 lung cancer dataset and several benchmark datasets, such as colon and ALL/AML. The results are displayed in Table 1. These experiments demonstrate that ClearF++ mostly outperforms (*p*-value < 0.05) other feature selection methods, such as MultiSURF [11], IFS [27], and ClearF [20], across the ARCHS4, colon, and ALL/AML datasets. ClearF++ achieved the highest performance across most of the feature subsets, reaching its best performance at 60 features both in the colon dataset (AUC = 0.826) and the ARCHS4 lung dataset (AUC = 0.983). Likewise, in the ALL/AML dataset, excluding the comparison with ClearF when the number of features was 60, ClearF++ outperformed other methods (*p*-value < 0.05), achieving the best performance at both 45 and 60 features (AUC = 0.949). Overall, these results highlight the consistent and enhanced performance of ClearF++ across varying numbers of features, showing the robustness and effectiveness of ClearF++. To show the statistical significance of the improvement, we included the results of a paired *t*-test between ClearF++ and other methods in Table S2 of the Supplementary Material, aligning with the results in Table 1. The results predominantly affirmed the notable superiority of our proposed method compared with other methods, with the exceptions of the case where ClearF++ vs. ClearF selected 60 features in the ALL/AML dataset, and the case where 45 features were selected in the colon dataset.

### 3.3. Performance Evaluation across Varying Feature and Sample Sizes

To assess the stability of our proposed method with a limited number of data samples, we evaluated lung cancer classification performance using only 5% of the training data samples from the ARCHS4 dataset. The number of features to be selected increased by 5, starting from 10, in accordance with the experimental procedure employed in the previous study [20].

**Figure 2:**
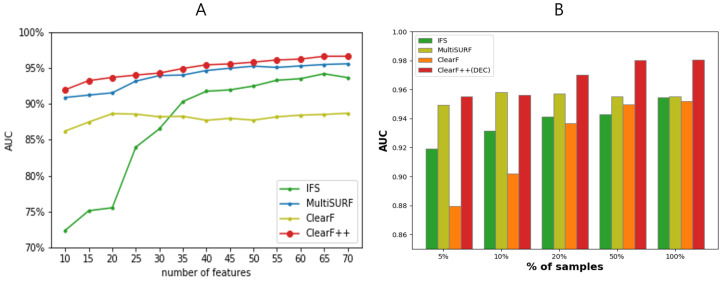
(**A**) Performance comparison across varying numbers of features between ClearF++ and other algorithms. The experiment was obtained using only 5% of the sample for training. (**B**) A comparative experiment measuring performances as the number of samples changes. The experiment was conducted by fixing the number of features at 45.

Figure 2 presents the experimental results on the ARCHS4 lung cancer dataset, using only 5% of the training samples. Our proposed method, ClearF++, exhibited superior performance compared with ClearF and other comparable feature selection algorithms. As displayed in Figure 2A, we observed that the stability of ClearF++ was preserved, while other methods yielded relatively unstable and poor performances with a limited number of samples.

In Figure 2B, we conducted experiments with varying numbers of samples to investigate the stability of ClearF++ across different sample sizes, fixing the number of features at 45. The experimental results reveal that ClearF++ and MultiSURF showed stable and improved performance across varying numbers of features, even with a small sample size. However, ClearF++ outperformed other methods when more than 10% of the samples were used, whereas MultiSURF exhibited no improvement when larger sample sizes were used and even suffered from slight performance degradation.

These results indicate that our proposed method, ClearF++, demonstrates impressive stability and performance even when dealing with a limited number of data samples. In comparison with other feature selection algorithms, ClearF++ consistently outperforms them, particularly when utilizing more than 10% of the training samples. This highlights the robustness and effectiveness of ClearF++ in various feature or sample size scenarios. These findings emphasize the potential of ClearF++ as a robust and effective feature selection technique, capable of maintaining its performance across a range of feature or sample sizes.

### 3.4. Effect of Feature-Wise Clustering Algorithms

**Figure 3:**
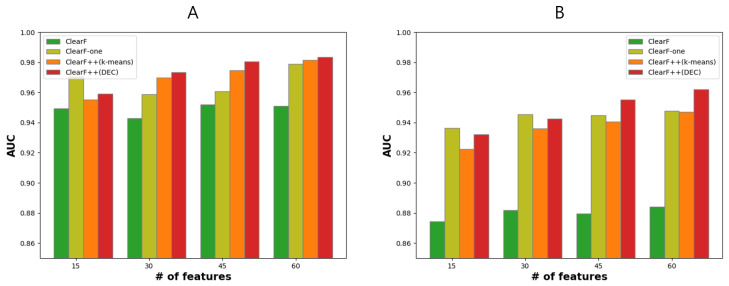
Performance evaluation of ClearF-based methods with various experimental settings in a lung cancer dataset. ClearF-one limits the number of bottleneck layers to 1, and ClearF++ applies feature-wise clustering in the proposed algorithm; thus, two clustering algorithms were compared. (**A**) Results using entire samples. (**B**) Results using 5% of samples for training.

We conducted an ablation study to confirm that our proposed method, ClearF++, indeed contributes to performance improvement compared with the previously proposed method, ClearF. For low-dimensional embedding, KernelPCA with an RBF kernel that showed the best performance in ClearF was utilized in ClearF-one and ClearF++. For the clustering method in ClearF++, k-means and DEC [21] were used. The ablation study was conducted on the ARCHS4 lung cancer dataset, with the results shown in Figure 3. Figure 3A displays the results for the entire samples, while Figure 3B presents the result using only 5% of the samples for training.

The experimental results show that ClearF-one, which substantially restricts the number of bottleneck layers, contributes to performance improvement, particularly when only a small portion of samples (5%) is used for training. This suggests that ClearF-one is effective in handling limited data samples and can still yield improved performance by simplifying the architecture, reducing the complexity, and focusing on the most relevant features. It is noteworthy that in the context of k-means clustering, the results yielded from training with only 5% of the samples shortly underperformed in comparison with those obtained from ClearF-one without clustering. Under the constraints of a small data size, k-means clustering appeared to struggle in achieving effective clustering. However, when we employed the more sophisticated clustering algorithm, DEC, we observed stable and enhanced performances when feature-wise clustering was applied. Particularly, DEC outperformed k-means clustering in terms of performance when a larger number of features were being selected, both in the scenarios with all samples in Figure 3A and with a small number of samples in Figure 3B. This suggests that DEC may be more suitable for handling complex data structures and capturing underlying patterns in the data when compared with the k-means clustering algorithm, particularly in situations with limited data samples.

**Figure 4:**
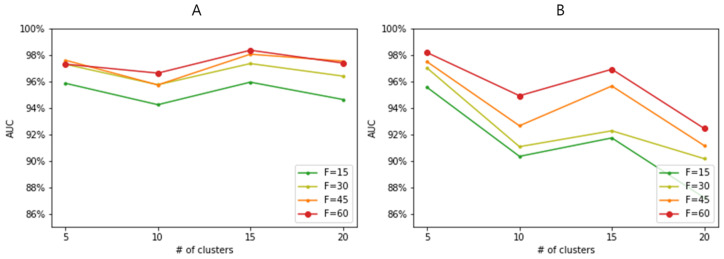
Performance evaluation based on varying numbers of clusters in the lung cancer dataset. F represents the number of selected features. (**A**) Performances of ClearF++ when DEC clustering is applied. (**B**) Performances of ClearF++ when k-means clustering is applied.

We evaluated ClearF++ performance by employing two different clustering methods with varying numbers of clusters. Figure 4A presents the results using the DEC clustering algorithm, while Figure 4B shows the results using k-means clustering. The results reveal that DEC with 15 clusters yielded the best performance, while k-means clustering achieved optimal results with 5 clusters. Although the best performances of these two clustering methods were comparable (close to AUC = 0.98), k-means clustering showed considerable variance depending on the number of clusters. In contrast, DEC demonstrated smaller variance and consistently higher performance across different cluster numbers. Consequently, while both clustering methods (DEC and k-means) can achieve comparable performances, DEC not only improved performances but showed more consistent and reliable results across different numbers of clusters. It suggests that the choice of clustering algorithm is important and that the proposed feature-wise clustering idea of ClearF++ contributes to more robust and improved performances.

### 3.5. Computational Cost Validation

To verify the computational efficiency of ClearF++ over other methods, we measured and compared the CPU time by running each method 10 times. The experiment utilized data containing 5000 randomly generated features and 5000 samples. As shown in Table 2, ClearF++ showed a substantially faster execution time than MultiSURF and IFS. While it showed a slightly slower execution time than ClearF due to the inclusion of clustering time, the improved performance ensures its competitiveness and effectiveness in comparison with other methods.

### 3.6. Functional Enrichment Analysis

To identify high-scoring features, we analyzed the ARCHS4 dataset using ClearF++, which demonstrated improved performance in our experiments. Since scores were calculated for each of the 10 folds, we defined an integrated scoring method. The top 100 features in each fold were assigned scores in descending order, ranging from 100 to 1 point. We then calculated the rank scores by averaging the scores obtained across all folds. The 50 highest-scoring genes are shown in Table S1. Furthermore, to investigate the biological relationships among the selected genes, we performed pathway and gene ontology enrichment analysis using ToppGene [28] on the top 50 genes. The results are shown in Table 3, respectively.

In biomarker detection, high classification accuracy of selected features does not guarantee that features associated with the disease are selected. Considering that the purpose of our algorithm is to select features for identifying important biomarkers, it is crucial to determine whether the top-scoring features are associated with the target disease. The 50 genes with high scores in our method are shown in Table S1. We performed enrichment analysis using these 50 genes.

The enrichment analysis results conducted with ToppGene [28] reveal that several genes related to the glycosaminoglycan (GAG) metabolism pathway received high scores. The extracellular matrix (ECM) regulates cell fate, and glycosaminoglycans (GAGs) are major macromolecules that compose the ECM, which play well-known roles in cancer angiogenesis, proliferation, invasion, and metastasis [29]. GAGs have been widely studied as treatments for cancer, inflammation, infection, and lung diseases, and one study [30] clarified the role of GAGs, contributing to future research.

Among the GAG-associated genes that received high scores in our method are GPC1, NDST1, CSPG4, and SDC3. An experiment involving CSPG4-specific mAb 225.28 demonstrated the regression induction of tumor metastasis in a lung metastasis model [31]. Endothelial cell (ECs) junction disassembly, a key step in inflammation, allows for vascular leakage during disease, and thrombin-cleaved fragments of the SDC3 ectodomain promote this process in human lung microvessels in certain cases [32]. NDST1 participates in the synthesis of the heparan sulfate (HS) chain of HSPG, and a study [33] found that it may provide an explanation for the clinical observation that heparin can improve outcomes in small-cell lung cancer (SCLC). Another study [34] suggested that NDST1 is associated with angiogenesis and tumor growth in lung tumors. There is also a study that recommended the use of glypican-1 (GPC1) as an additional positive marker for lung squamous cell carcinoma [35]. These findings suggest that analyzing the effects of NDST1 and SDC3 expression on pulmonary blood vessels in relation to GAGs may be helpful in diagnosing and treating lung cancer.

Additionally, enrichment analysis results using the top 50 genes with high scores (Table 3) reveal that 7 genes related to the collagen metabolic process are included in the biological process. These genes are P3H3, MRC2, MMP14, ENG, EMILIN1, CREB3L1, and COL1A1, with CREB3L1, EMILIN1, and COL1A1 ranking 1st, 3rd, and 4th, respectively. Impaired collagen metabolism is accompanied by increased prolidase activity in lung cancer squamous epithelium [36]. Furthermore, idiopathic pulmonary fibrosis (IPF) is associated with an increased risk of lung cancer with elevated collagen and prolidase activity [36,37,38]. On the other hand, Prolidase Deficiency (PD) and osteogenesis imperfecta (OI) share similar phenotypes [37]. Notably, our enrichment analysis includes CREB3L1 and COL1A1, which, out of the top 10 genes, are associated with osteogenesis imperfecta type III (disorder). The expression of β1 integrin, which has been shown to regulate prolidase activity, is decreased in OI [37,39,40]. However, there is no difference in the levels of β1 integrin between healthy lung cells and cancer cells, suggesting that prolidase regulation in lung cancer may involve a different mechanism [36,37]. Therefore, studying the role of prolidase, CREB3L1, and COL1A1 gene expression in lung cancer appears to be significant.

In addition to the aforementioned genes, we found several high-scoring genes (shown in Table S1) that have been linked to lung cancer in multiple studies. The CREB3L1 gene has been associated with lung cancer growth due to its involvement in the activation of alpha-smooth muscle actin (α-SMA)-positive cancer-associated fibroblasts (CAFs) [41]. SYDE1 is associated with epithelial–mesenchymal transition (EMT) reversal, which is associated with the progression of various tumors, including lung cancer [42]. Reduced EMILIN-1 production in some tumor types is associated with higher proliferation of tumor cells in breast and lung cancer [43]. Another study [44] suggested that COL1A1 can be a potential biomarker for poor progression-free survival and chemoresistance in metastatic lung cancer. Serum CKAP4 levels can distinguish lung cancer patients from healthy controls, making it a potential serum diagnostic marker for lung cancer [45]. Carbohydrates associated with LAMP1 play a crucial role in determining lung metastasis [46]. A potential target of TAF15 concerning resistance to radiotherapy, essential for non-small-cell lung cancer treatment, has been proposed [47]. TBX2 subfamily methylation may serve as a potential biomarker for early detection and intervention in non-small-cell lung cancer [48]. Consequently, the genes selected by our method are shown to be biomarker candidates for lung cancer.

## 4. Discussion

We evaluated the suitability of our methodology for biomarker detection from a machine learning perspective. The results in Figure 2 demonstrate that our proposed method is effective when selecting a small number of features. Particularly, when combined with Figure 3, ClearF-one generally yields favorable results when selecting from 10 to 25 features, and ClearF++ shows improvement when selecting from 30 to 50 features. Given the importance of selecting a small number of features in biomarker discovery, our method can be considered suitable. Moreover, our approach demonstrates stable performance even with a small sample size, as shown in Figure 2B. In the biomedical field, insufficient learning samples are often encountered, and our method proves effective in such cases. Additionally, as shown in Table 2, our method can be effectively employed in environments with limited computational power due to its advantageous execution time.

The results in Figure 3 indicate that ClearF++ can show degraded performances compared with the model without clustering (ClearF-one) when the number of features is small. This is likely because ClearF-one effectively selects a small number of features when only certain information remains after embedding the entirety of the data into a single bottleneck layer. However, when the number of features increases, it suffers from performance degradation due to information loss. In contrast, ClearF++ extracts information for each cluster, which provides more robust and improved performances when selecting multiple features.

Our method addresses the sensitivity issue related to the number of bottleneck layers in the previously proposed method, ClearF, but still requires many parameter adjustments. In particular, determining the number of clusters remains a challenge in clustering. Although Figure 4 shows that our method is not highly sensitive to the number of clusters within a range of 5 to 20 clusters, an exceptionally higher number of clusters, such as 50 or 100, led to instability in clustering results and a substantial performance decrease. Through our experiments, we discerned that the optimal number of clusters likely resides within the 5 to 20 range. However, this range may vary with different datasets according to their sample sizes. Accordingly, future research could focus on the automatic selection of the number of clusters. Further, there is still an issue in setting the model structure or learning method in the part that utilizes DEC. This issue can be addressed in future studies. Furthermore, our method exhibits flexibility towards a range of clustering algorithms. Our experimental findings, as illustrated in Figure 3, indicated that employing a more sophisticated clustering technique could result in more stable and improved performances. Thus, future research that utilizes advanced clustering methods could potentially enhance performances.

## 5. Conclusions

In this study, we developed an improved feature selection algorithm for identifying biomarkers that can be used for disease prediction and biomedical data analysis. Our experimental results demonstrate several advantages of our method, including improved prediction performance and faster execution Furthermore, it shows substantially stable performance even with a limited number of samples, making it particularly effective for biomedical data analysis, where the available sample size is often insufficient.

One limitation of our method is that it requires determining the optimal number of clusters, which can vary across different datasets. In this study, we experimented with several scenarios to select the most appropriate parameters. However, automatic parameter selection methods can be exploited in future work to address this issue.

## Figures and Tables

**Table 1:** Performance comparison on several benchmark datasets across a varying number of features. The performance was measured with the average AUC of 10-fold cross-validation.

n ^1^	Colon				ALL/AML				ARCHS4			
	MultiSURF	IFS	ClearF	ClearF++	MultiSURF	IFS	ClearF	ClearF++	MultiSURF	IFS	ClearF	ClearF++
15	0.648	0.749	0.707	**0.805**	0.906	0.913	0.912	**0.947**	0.925	0.927	0.949	**0.959**
30	0.711	0.672	0.765	**0.773**	0.927	0.926	0.921	**0.940**	0.944	0.953	0.943	**0.973**
45	0.703	0.658	**0.801**	0.751	0.938	0.915	0.927	**0.949**	0.955	0.955	0.952	**0.980**
60	0.723	0.761	0.815	**0.826**	0.927	0.915	**0.949**	**0.949**	0.969	0.949	0.951	**0.983**

^1^ The number of features.

**Table 2:** Computational costs comparison of ClearF++ and other feature selection methods.

Methods	IFS	MultiSURF	ClearF	ClearF++
CPU times (s)	398.91 ± 17.22	78,515.59 ± 346.53	132.68 ± 6.51	166.85 ± 10.77

**Table 3:** Pathway and gene ontology enrichment analysis results using ToppGene on the top 50 ranked genes. The 10 most significant gene ontology (GO) terms that have the lowest p-values are shown, as well as pathway and disease terms with significant *p*-values (p<0.05) from the enrichment analysis.

Category	ID	Name	*p*-Value	q-Value ^1^	q-Value ^2^	HC ^3^	HCG ^4^
BP	GO:0032963	collagen metabolic process	$2.89\times 10^{-8}$	$5.13\times 10^{-5}$	$5.13\times 10^{-5}$	7	144
BP	GO:0030042	actin filament depolymerization	$5.03\times 10^{-7}$	$8.95\times 10^{-4}$	$3.68\times 10^{-4}$	5	71
BP	GO:0032964	collagen biosynthetic process	$6.63\times 10^{-7}$	$1.18\times 10^{-3}$	$3.68\times 10^{-4}$	5	75
MF	GO:0044877	protein-containing complex binding	$7.35\times 10^{-7}$	$2.09\times 10^{-4}$	$2.09\times 10^{-4}$	16	1726
BP	GO:0001568	blood vessel development	$8.27\times 10^{-7}$	$1.47\times 10^{-3}$	$3.68\times 10^{-4}$	13	1152
BP	GO:0035904	aorta development	$1.47\times 10^{-6}$	$2.62\times 10^{-3}$	$4.71\times 10^{-4}$	5	88
BP	GO:0001944	vasculature development	$1.87\times 10^{-6}$	$3.33\times 10^{-3}$	$4.71\times 10^{-4}$	13	1239
BP	GO:0030198	extracellular matrix organization	$2.21\times 10^{-6}$	$3.93\times 10^{-3}$	$4.71\times 10^{-4}$	8	394
BP	GO:0043062	extracellular structure organization	$2.25\times 10^{-6}$	$4.00\times 10^{-3}$	$4.71\times 10^{-4}$	8	395
BP	GO:0045229	external encapsulating structure organization	$2.38\times 10^{-6}$	$4.24\times 10^{-3}$	$4.71\times 10^{-4}$	8	398
Disease	C0268362	Osteogenesis imperfecta type III (disorder)	$1.90\times 10^{-6}$	$3.42\times 10^{-3}$	$3.42\times 10^{-3}$	3	11
Pathway	1269980	Heparan sulfate/heparin (HS-GAG) metabolism	$1.82\times 10^{-5}$	$5.99\times 10^{-3}$	$1.82\times 10^{-3}$	4	54
Pathway	1309217	Defective B3GALT6 causes EDSP2 and SEMDJL1	$2.21\times 10^{-5}$	$7.26\times 10^{-3}$	$1.82\times 10^{-3}$	3	19
Pathway	1269015	Defective B3GAT3 causes JDSSDHD	$2.21\times 10^{-5}$	$7.26\times 10^{-3}$	$1.82\times 10^{-3}$	3	19
Pathway	1269014	Defective B4GALT7 causes EDS, progeroid type	$2.21\times 10^{-5}$	$7.26\times 10^{-3}$	$1.82\times 10^{-3}$	3	19
Pathway	1269981	A tetrasaccharide linker sequence is required for GAG synthesis	$5.84\times 10^{-5}$	$1.92\times 10^{-2}$	$3.20\times 10^{-3}$	3	26
Pathway	1269011	Diseases associated with glycosaminoglycan metabolism	$5.84\times 10^{-5}$	$1.92\times 10^{-2}$	$3.20\times 10^{-3}$	3	26
Pathway	1269982	HS-GAG biosynthesis	$9.99\times 10^{-5}$	$3.29\times 10^{-2}$	$4.69\times 10^{-3}$	3	31
Pathway	M39870	Type I collagen synthesis in the context of osteogenesis imperfecta	$1.21\times 10^{-4}$	$3.97\times 10^{-2}$	$4.97\times 10^{-3}$	3	33
Pathway	1268756	Unfolded Protein Response (UPR)	$1.48\times 10^{-4}$	$4.88\times 10^{-2}$	$5.42\times 10^{-3}$	4	92

^1^ Bonferroni q-value, ^2^ FDR B&H q-value, ^3^ Hit Count in the query list, ^4^ Hit count in the genome.

## Data Availability

All data that are not presented in the main paper are available from the corresponding author on request.

## Supplementary Materials

The following supporting information can be downloaded at: https://www.mdpi.com/article/10.3390/bioengineering10070824/s1, Table S1: List of the highest-scoring 50 genes in the ARSCH4 lung cancer dataset; Table S2: Results of statistical significance tests between ClearF++ and three other methods: MultiSURF, IFS, and ClearF, corresponding to the results presented in Table 1. The *p*-value was measured through a paired *t*-test between ClearF++ and other methods across three different datasets.
