# Supplementary material for: ClearF++: Improved Supervised Feature Scoring Using Feature Clustering in Class-Wise Embedding and Reconstruction
Source: Bioengineering (Basel). 2023 Jul 10;10(7):824. doi: 10.3390/bioengineering10070824 (PMC10376817; doi:10.3390/bioengineering10070824)
Supplement: Supplementary file 1 [file bioengineering-10-00824-s001.zip › bioengineering-2432642--supplementary.pdf]

Supplementary Materials: ClearF++: Improved Supervised Feature Scoring using Feature Clustering in Class-wise Embedding and Reconstruction

Sehee Wang [1], So Yeon Kim [1,2] and Kyung-Ah Sohn [1,2]*

1. Supplementary Table

Table S1. List of the highest-scoring 50 genes in the ARSCH4 lung cancer dataset.

Rank	Gene symbol	Rank score	Gene Description
1	CREB3L1	99.2	CAMP Responsive Element Binding Protein 3 Like 1
2	SYDE1	95.7	Synapse Defective Rho GTPase Homolog 1
3	EMILIN1	93.0	Elastin Microfibril Interfacer 1
4	COL1A1	89.9	Collagen Type I Alpha 1 Chain
5	SEC61A1	88.8	SEC61 Translocon Subunit Alpha 1
6	CKAP4	87.0	Cytoskeleton Associated Protein 4
7	LAMP1	84.2	Lysosomal Associated Membrane Protein 1
8	TAF15	84.1	TATA-Box Binding Protein Associated Factor 15
9	CSPG4	83.4	Chondroitin Sulfate Proteoglycan 4
10	TBX2	82.6	T-Box Transcription Factor 2
11	ADD1	81.5	Adducin 1
12	MXRA8	77.9	Matrix Remodeling Associated 8
13	PTRF	76.9	Caveolae Associated Protein 1
14	NUBP2	76.6	NUBP Iron-Sulfur Cluster Assembly Factor 2, Cytosolic
15	MRC2	76.4	Mannose Receptor C Type 2
16	ENG	74.5	Endoglin
17	MYH9	73.4	Myosin Heavy Chain 9
18	POFUT2	72.9	Protein O-Fucosyltransferase 2
19	TSTA3	72.4	GDP-L-Fucose Synthase
20	DBN1	70.6	Drebrin 1
21	CAPG	68.6	Capping Actin Protein, Gelsolin Like
22	WDR1	67.6	WD Repeat Domain 1
23	BSCL2	67.4	BSCL2 Lipid Droplet Biogenesis Associated, Seipin
24	FAM129B	67.2	Niban Apoptosis Regulator 2
25	C12orf10	64.7	MYG1 Exonuclease
26	MAP4	63.4	Microtubule Associated Protein 4
27	DPAGT1	61.5	Dolichyl-Phosphate N-Acetylglucosaminephosphotransferase 1
28	MYO9B	61.3	Myosin IXB
29	CD320	59.4	CD320 Molecule
30	CDC42SE2	59.4	CDC42 Small Effector 2
31	SDC3	56.4	Syndecan 3
32	EXOSC4	55.3	Exosome Component 4
33	FKBP10	55.1	FKBP Prolyl Isomerase 10
34	MMP14	55.1	Matrix Metallopeptidase 14
35	MFSD5	54.1	Major Facilitator Superfamily Domain Containing 5
36	SCPEP1	53.4	Serine Carboxypeptidase 1
37	LOXL1	51.2	Lysyl Oxidase Like 1
38	ATP5D	50.4	ATP Synthase F1 Subunit Delta
39	FTSJ1	49.9	FtsJ RNA 2′-O-Methyltransferase 1
40	CALR	49	Calreticulin

Continued on next page

Table S1 – continued from previous page

Rank	Gene symbol	Rank score	Gene Description
41	SPNS1	47.3	SPNS Lysolipid Transporter 1, Lysophospholipid
42	DCXR	45.8	Dicarbonyl And L-Xylulose Reductase
43	WDR18	45.1	WD Repeat Domain 18
44	MICAL2	44.8	Microtubule Associated Monooxygenase, Calponin And LIM Domain Containing 2
45	ARHGEF17	43.3	Rho Guanine Nucleotide Exchange Factor 17
46	NDST1	43.2	N-Deacetylase And N-Sulfotransferase 1
47	GPC1	41.8	Glypican 1
48	DPM2	41.2	Dolichyl-Phosphate Mannosyltransferase Subunit 2, Regulatory
49	METTL13	40.5	Methyltransferase 13, EEF1A Lysine And N-Terminal Methyltransferase
50	P3H3	40	Prolyl 3-Hydroxylase 3

Table S2. Results of statistical significance tests between ClearF++ and three other methods: MultiSURF, IFS and ClearF, corresponding to the results presented in Table 1. The p-value was measured through a paired t-test between ClearF++ and other methods across three different datasets.

n[1]	Colon			ALL/AML			ARCHS4		
	ClearF++ vs. MultiSURF	IFS	ClearF	ClearF++ vs. MultiSURF	IFS	ClearF	ClearF++ vs. MultiSURF	IFS	ClearF
15	2.43E-10	2.22E-06	5.74E-09	5.46E-05	1.00E-05	2.24E-05	8.73E-07	5.24E-06	6.26E-04
30	2.41E-08	1.41E-11	1.30E-01	2.46E-03	3.46E-02	6.56E-05	3.06E-05	1.02E-03	7.02E-07
45	4.15E-06	3.52E-07	5.67E-07	6.62E-03	9.36E-08	1.29E-05	9.30E-07	9.39E-08	7.22E-06
60	3.14E-13	2.54E-12	2.19E-03	6.23E-04	2.69E-05	1.00E+00	4.64E-04	1.03E-06	8.48E-08

[1] The number of features.
